# Down-regulation of miRNA-148a and miRNA-625-3p in colorectal cancer is associated with tumor budding

**DOI:** 10.1186/s12885-017-3575-z

**Published:** 2017-09-01

**Authors:** Edita Baltruskeviciene, Diana Schveigert, Vaidotas Stankevicius, Ugnius Mickys, Tadas Zvirblis, Jaroslav Bublevic, Kestutis Suziedelis, Eduardas Aleknavicius

**Affiliations:** 1 Departament of medical oncology, National Cancer Institute, Santariskiu 1, 08660 Vilnius, LT Lithuania; 2 Laboratory of Molecular Oncology, National Cancer Institute, Vilnius, Lithuania; 3 Institute of Biotechnology, Life Sciences Center, Vilnius University, Vilnius, Lithuania; 4 National Center of Pathology, affiliate of Vilnius University Hospital Santaros Klinikos, Vilnius, Lithuania; 5 Hematology, oncology and transfusiology center, Vilnius University Hospital Santaros Klinikos, Vilnius, Lithuania; 6 Institute of Biosciences, Life Sciences Center, Vilnius University, Vilnius, Lithuania; 7 Department of Radiology, Nuclear Medicine and Physics of Medicine, Faculty of Medicine, Vilnius University, Vilnius, Lithuania

**Keywords:** MiRNA-148a, miRNA-625-3p, microRNA, Tumor budding, Colorectal cancer, Oxaliplatin

## Abstract

**Background:**

MiRNAs are often deregulated in colorectal cancer and might function as tumor suppressors or as oncogenes. They participate in controlling key signaling pathways involved in proliferation, invasion and apoptosis and may serve as prognostic and predictive markers. In this study we aimed to evaluate the role of miRNA-148a and miRNA-625-3p in metastatic colorectal cancer.

**Methods:**

Fifty-four patients with a first-time diagnosed CRC receiving FOLFOX ± Bevacizumab were involved in the study. Tumor samples underwent routine pathology examination including evaluation for tumor budding and KRAS. MiRNA-148a and miRNA-625-3p expression analysis was done by RT-PCR. Associations between expression of both miRNAs and clinico-pathological factors, treatment outcomes and survival were analyzed.

**Results:**

Both miRNA-148a and miRNA-625-3p were down-regulated in the tumors compared to normal colonic mucosa. Significantly lower expression of both miRNAs was noticed in tumors with budding phenomenon compared to tumors without it (median values of miRNA-148a were 0.314 and 0.753 respectively, *p* = 0.011, and 0.404 and 0.620 respectively for miRNA-625-3p, *p* = 0.036). Significantly lower expression of miRNA-625-3p was detected in rectal tumors, compared to tumors in the colon (median 0.390 and 0.665 respectively, *p* = 0.037). Progression free survival was significantly lower in patients with high miRNA-148a expression (6 and 9 months respectively, *p* = 0.033), but there were no significant differences in PFS for miRNA-625-3p and in overall survival for both miRNAs.

**Conclusions:**

There was a significant relationship between low miRNA-148a and miRNA-625-3p expression and tumor budding, which is thought to represent epithelial-mesenchymal transition. Both studied miRNAs may be associated with a more aggressive phenotype and could be the potential prognostic and predictive biomarkers in CRC. Further investigation is needed to confirm miRNAs involvement in EMT, and their prognostic and predictive value.

**Electronic supplementary material:**

The online version of this article (10.1186/s12885-017-3575-z) contains supplementary material, which is available to authorized users.

## Background

Colorectal cancer (CRC) is one of the most frequently occurring cancers worldwide, accounting for 1.2 million new diagnoses and 600.000 deaths every year [1]. There is a tendency of decreasing morbidity and mortality, which could be associated with the development of new effective anticancer agents and cancer prophylactics. Identification of novel biomarkers, based on molecular changes caused by the disease, could help to improve those results. In combination with pathological and clinical evaluation, recent biomarker investigations involve genomic, proteomic and transcriptomic research.

Currently, prognosis and treatment choice is based mainly on the information regarding tumor stage. Only a few tumor markers (RAS and BRAF mutation, mismatch repair status) have been implemented into clinical practice [2, 3]. Recent evidence suggests, that tumor budding, described as the presence of individual cells and small clusters of tumor cells at the invasive front of the carcinoma, could serve as an additional prognostic factor in stage II CRC [4, 5], but there is little information regarding its significance in the advanced disease. The down-regulation of epithelial and the up-regulation of mesenchymal markers suggests that tumor budding is a morphological expression of epithelial mesenchymal transition (EMT), which is associated with tumor invasiveness, formation of metastases and resistance to chemotherapy [6, 7].

MicroRNAs (miRNAs) are a class of small (18–25 nucleotides in length), single-stranded noncoding RNAs. They negatively regulate the expression of target genes by binding to 3‘UTR segments of messenger RNAs and are aberrantly expressed in most human cancers, including CRC, in which they may function as tumor suppressors or as oncogenes [8]. This posttranscriptional regulation plays a crucial role in controlling key signaling pathways of CRC (RAS/RAF/MAPK, AKT/MTOR and others), though miRNAs are actively involved in proliferation, invasion, angiogenesis, apoptosis and other biological processes [8–10].

Studies examining miRNA expression in CRC have consistently reported the dysregulated expression of nearly 100 miRNAs, compared to non cancerous tissue. Among these, miRNA-143, miRNA-145, let-7, miRNA-148a, miRNA-20a, miRNA-21, miRNA-106, miRNA-155, and miRNA-203 in CRC were among the most frequently mentioned in the literature [11–14]. The expression of miRNAs in CRC tissue and blood samples could have a prognostic and predictive value [11, 15]. Also, an association between miRNA signature and metastases was reported [16].

We have focused on miRNA-148a and miRNA-625-3p because of the growing evidence of their importance in carcinogenesis, invasion and progression of CRC and because both of these miRNAs are associated with resistance to oxaliplatin based regimens [17–19]. Recent data shows that these miRNAs potentially regulate EMT through their targets such as MET/SNAIL, WNT signaling pathways, E-cadherin, N-cadherin, fibronectin and others [20–22].

## Methods

### Patients

Fifty-four patients with first-time diagnosed metastatic colorectal cancer participated in a prospective observational study conducted in the National Cancer Institute (Lithuania) in 2011–2014. The median age was 63 years (range 44–76). The male-female gender ratio of the group was 52% and 48%, respectively. 64% of the tumors were located in the colon and 36% in the rectum. The histological tumor type in 80% of the cases was adenocarcinoma, and in 20% – mucinous adenocarcinoma. 98% of the tumors were medium grade. All the patients had metastases in the liver, and for 38% of the patients it was the only site where metastases were detected. In 91% of the cases, the primary tumor was removed before starting chemotherapy. 25% of the patients had undergone resection or radiofrequency ablation of liver metastases and it was performed after 2 months of chemotherapy. The patients received FOLFOX4 chemotherapy with or without Bevacizumab until disease progression or an unacceptable toxicity, according to the institutional guidelines. The median number of chemotherapy cycles was 8. Bevacizumab was administered to 53% of the patients. Treatment efficacy was evaluated every 2 months by a CT (computer tomography) scan according to RECIST 1.1 criteria. After completing the treatment, patients were followed up for progression or survival every 3 months.

The study has been approved by the Regional Biomedical Research Ethics Committee and performed in accordance with the Helsinki declaration. All patients signed the informed consent form before entering the study. Tumor samples were analyzed in the National Pathology Center (Lithuania) and the Scientific Research Center of the National Cancer Institute (Lithuania), and testers were blinded to treatment allocation and outcomes.

### Pathology examination

A routine pathology examination, including KRAS testing, was performed. Additionally, samples were evaluated for tumor budding. All the pathology testing was performed by blinded pathologist before starting the treatment under study.

#### Tumor budding examination

Optimal tumor block for tumor budding evaluation was selected after reviewing H&E stained slides. Then H&E and pan-cytokeratin AE1/AE3 stained sections were assessed using the 10-HPF method. Areas of highest budding density were identified at low power field (×4-×10) and then tumor buds were counted under high magnification (×40). The cut-off value for determining a high degree of budding was considered 10 budding cells per high power field [23].

### MiRNA extraction and analysis

Formalin-fixed paraffin embedded tissue samples of primary tumors (ensuring the presence of at least 50% of tumour cells in the tumor) and adjacent normal mucosa were prepared by the pathologist. 4–5 sections of 5 μm thickness were obtained and processed for MiRNAanalysis. MiRNA from the samples was extracted using “miRNeasy FFPE Kit” (QIAGEN, Germany). The concentration of miRNA was measured by “NanoDrop2000C” spectrophotometer (Thermo Fisher Scientific,USA). MiRNA expression analysis was done by the RT-PCR method using “TaqMan® MicroRNA Reverse Transkription Kit” (Life Technologies, USA) and “TaqMan® Universal Master Mix II, no UNG” (Life technologies, USA). Each sample was examined in triplicate. Changes in miRNA expression were calculated using the 2^-ΔΔCt^method.

### Bioinformatics

In silico miRNR target analysis was performed using microT-CDS algorithm and miRTarBase to extract theoretically predicted and experimentally validated target-genes, respectively [24, 25]. KEGG pathway enrichment analysis of obtained miRNA targets was completed using WebGestalt online tool kit (http://www.webgestalt.org/option.php) as described previously [26]. *P* values were evaluated using Hypergeometric distribution and adjusted with Benjamini and Hochberg post-hoc test. Genes involved in epithelial-mesenchymal transition (EMT) were retrieved form dbEMT database. Genes potentially regulated by miR-148a were further visualized using Cytoscape ver. 3.4.0.

### Statistical analysis

Descriptive statistics were used to describe demographic characteristics. The non-parametric Wilcoxon test was used to evaluate the differences between the two data sets. The differences between the two qualitative data groups were evaluated by the Chi-square test. Risk factors for PFS and OS were assessed by Cox regression analysis. Survival trends were evaluated by the Kaplan-Meier method. A log-rank test was used to evaluate the difference between Kaplan-Meier curves. Progression free survival (PFS) was calculated as the timespan from the first day of treatment to the first date of disease progression, the day of a confirmed new tumor or death. Overall survival (OS) was calculated as the time from the first day of treatment to death. If during the last visit to the clinician there was no evidence of disease progression or a new tumor, the date was confirmed as censored. A two-tailed *p*-value less than 0.05 was considered to be significant. Statistical analysis was performed using the Statistical Analysis System (SAS) package version 9.2.

## Results

**Fig. 1:**
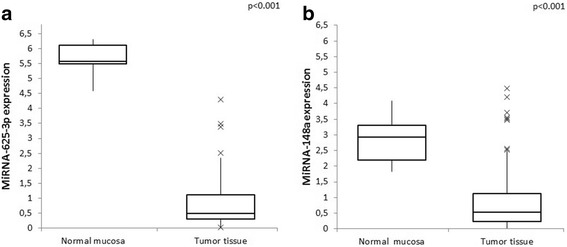
Differences in miRNA-148a **a** and miRNA-625-3p **b** expression in tumor tissue and normal adjacent mucosa

Both miRNA-148a and miRNA-625-3p were down-regulated in the tumors compared to normal adjacent mucosa. The median values of miRNA-148a were 0.527 (range 0.005–4.485) and 2.94 (range 1.826–4.091) respectively, *p* < 0.001, and for miRNA-625-3p – 0.492 (range 0.021–4.289) and 5.584 (4.595–6.314) respectively, *p* < 0.001 (Fig. 1). The patients were divided into high and low expression groups based on the median value. There were no significant differences in the groups according to the age, gender, number and location of metastases or KRAS mutations (Table 1). A significantly higher number of tumors in the colon had a higher expression of miRNA-625-3p (*p* = 0.024), but there were no association between miRNA-148a expression and tumor location. There was a trend toward lower miRNA-148a and miRNA-625-3p expression in tumors with a budding phenomenon (both *p* = 0.051).

**Table 1:** The association of miRNA-148a, miRNA-625-3p and tumor budding with clinical and pathological characteristics

Characteristic	N	miRNA 148-a expression			miRNA 625-3p expression			Tumor budding		
		Low	High	p	Low	High	p	Yes	No	p
Total	54	27	27		27	27		21	33	
Age										
< 65	28	17	12	0.172	15	13	0.586	14	15	0.128
≥ 65	26	10	15		12	14		7	18	
Gender										
Female	26	13	13	1.000	13	13	1.000	12	14	0.291
Male	28	14	14		14	14		9	19	
Location										
C18–19	34	15	19	0.260	13	21	0.024	11	23	0.199
C20	20	12	8		14	6		10	10	
Side										
Right	12	6	6	1.000	5	7	0.513	7	5	0.117
Left	42	21	21		22	20		14	28	
T										
1–3	36	17	19	0.564	16	20	0.248	14	22	1.000
4	18	10	8		11	7		7	11	
N										
0–1	34	15	19	0.260	16	18	0.573	11	23	0.199
2	20	12	8		11	9		10	10	
Extrahepatic metastases										
Yes	33	15	19	0.260	15	18	0.402	14	19	0.402
No	21	12	8		12	9		7	14	
Number of metastatic sites										
1–2	39	20	19	0.762	22	17	0.129	15	24	0.917
≥ 3	15	7	8		5	10		6	9	
Type of adenocarcinoma										
Adenocarcinoma	43	24	19	0.091	24	19	0.311	18	25	0.376
Mucinous	11	3	8		4	7		3	8	
Tumor budding										
Yes	21	14	7	0.051	14	7	0.051	-	-	-
No	33	13	20		13	20		-	-	
KRAS										
Mutated	37	18	19	0.770	17	20	0.379	15	22	0.713
Wild type	17	9	8		10	7		6	11	

**Fig. 2:**
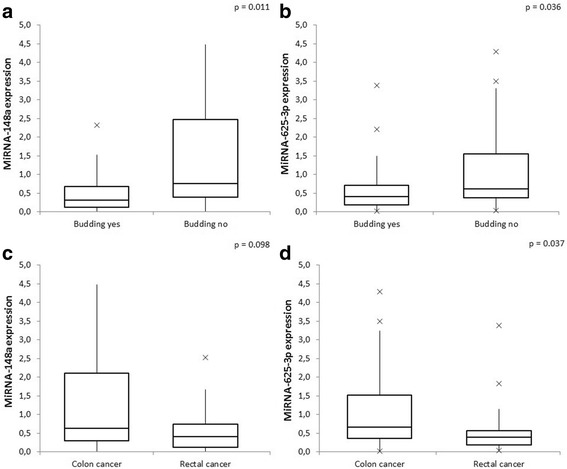
Differences in miRNA-148a and miRNA-625-3p expression in accordance to tumor budding and tumor location. **a**. MiRNA-148a expression according to tumor budding; **b**. MiRNA-625-3p expression according to tumor budding; **c**. MiRNA-625-3p expression according to tumor location; **d** MiRNA-625-3p expression according to tumor location

Further analysis revealed a significantly lower expression of both miRNAs in tumors with budding phenomenon compared to tumors without it (median values of miRNA-148awere 0.314 and 0.753 respectively, *p* = 0.011; and of miRNA-625-3p  were 0.404 and 0.620 respectively, *p* = 0.0360 (Fig. 2a and b). A lower expression of both miRNAs was also noticed in tumors of the rectum, compared to tumors in the colon (median values of miRNA-148a were 0.412 and 0.624 respectively, *p* = 0.098; and of miRNA-625-3p were 0.390 and 0.665 respectively, *p* = 0.037). The difference was significant only for miRNA-625-3p (Fig. 2c and d).

Based on treatment response, patients were divided into 2 groups: responders (complete and partial response; 32 patients, 58%), and non-responders (stable and progressive disease; 23 patients, 42%). There were no differences in response rates regarding miRNA-148a or miRNA-625-3p expression.

**Fig. 3:**
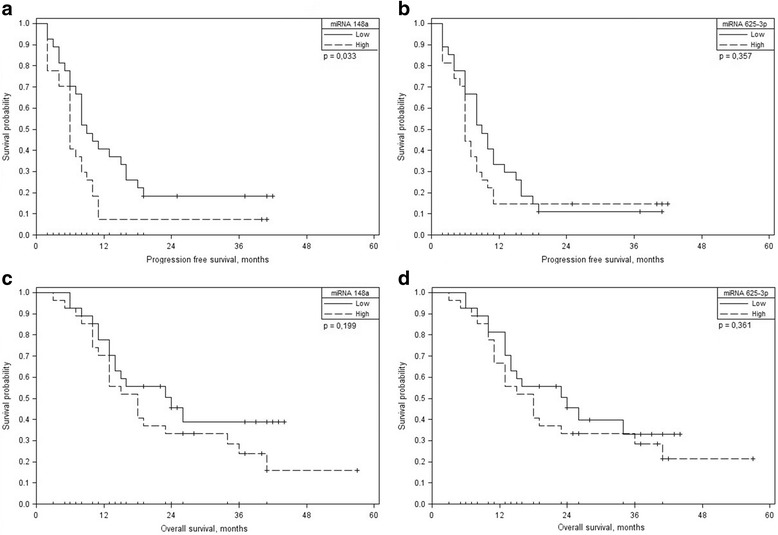
Influence of miRNA-148a and miRNA-625-3p expressionon OS and PFS. **a** Kaplan-Meier curves for PFS according to miRNA-148a expression; **b** Kaplan-Meier curves for PFS according to miRNA-625-3p expression; **c** Kaplan-Meier curves for OS according to miRNA-148a expression; **d** Kaplan-Meier curves for OS according to miRNA-625-3p expression

The median follow up was 16 months (range 3–51) for all patients. Progression free survival (PFS) was 6 (95% CI: 5–7) and 9 (95% CI: 6–12) months in patients with high and low miRNA-148a expression respectively (*p* = 0.033); and 6 (95% CI: 5–7) and 9 (95% CI: 6–12) months in patients with high and low miRNA-625-3p expression respectively (*p* = 0.357) (Fig. 3a and b).

The overall survival (OS) was 18 (95% CI: 12–24) and 24 (95% CI: 18–30) months in patients with high and low miRNA-148a expression respectively (*p* = 0.199); and 18 (95% CI: 12–24) and 24 (95% CI: 18–30) months in patients with high and low miRNA-625-3p expression, respectively (*p* = 0.361) (Fig. 3c and d).

The Univariate Cox regression analysis revealed that a higher expression of miRNA-148a was associated with a shorter PFS. No other significant factors were found in this analysis.

Budding phenomenon was detected in 39% of cases. There were no significant differences in the groups with budding compared to no budding and age, sex, location of the tumor, size, lymph node involvement, histological type, metastases or KRAS mutation (Table 1). In further analysis, the budding status did not influence PFS – 7 (95% CI: 5–9) and 8 (95% CI: 5–11) months respectively (*p* = 0.499); or OS – 18 (95% CI: 5–31) and 18 (95% CI: 7–29) month respectively (*p* = 0.709).

**Fig. 4:**
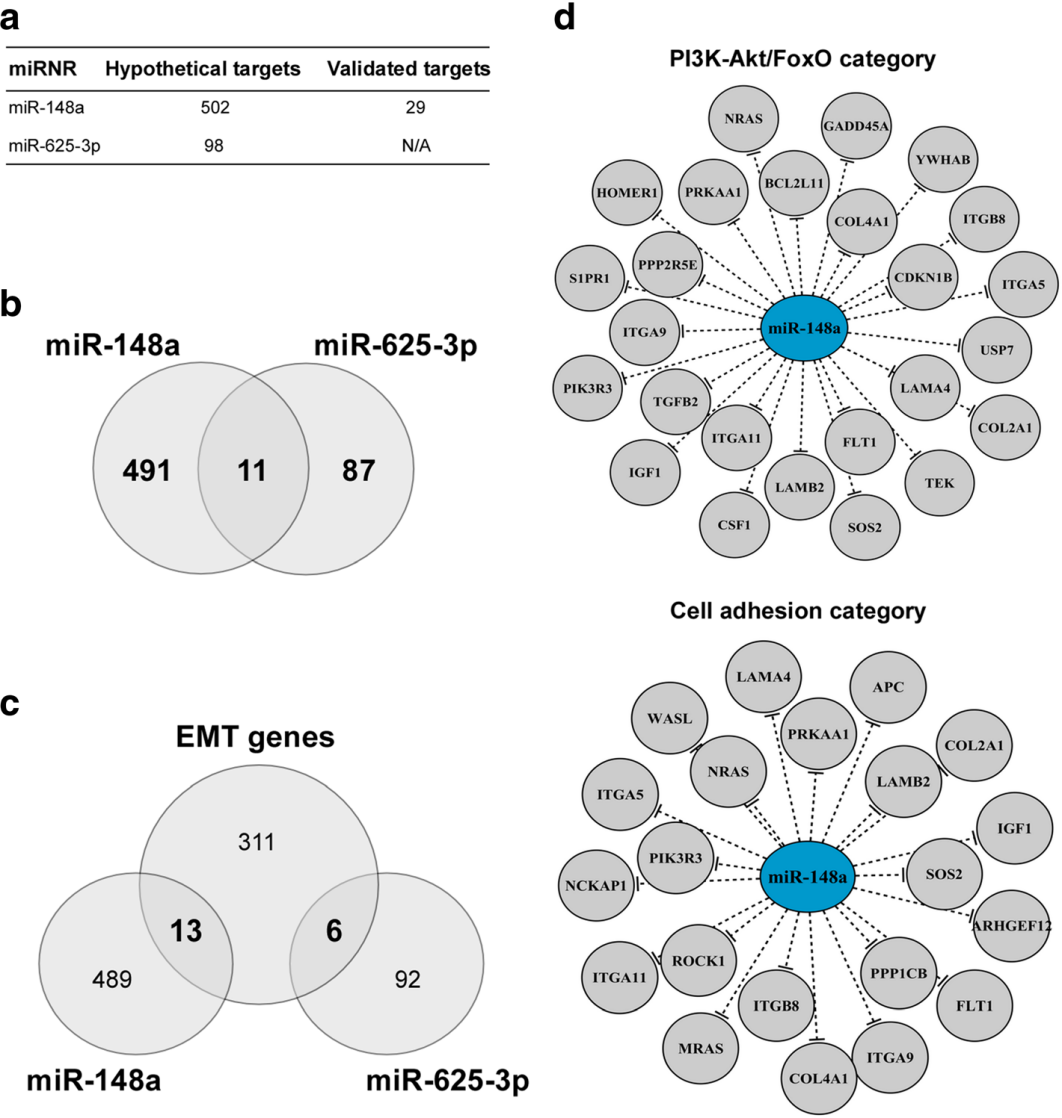
miR-148a and miR-625-3p target analysis. **a**. Table depicting hypothetical and validated target-genes of miR-148a and miR-625-3p miRNAs; **b**. Venn diagram showing the overlap between miR148a and miR-625-3p target-genes; **c**. Venn diagram showing the overlap between miR148a/miR-625-3p target-genes and genes involved in epithelial-mesenchymal transition (EMT); **d**. miR-148a-gene network visualizing target-genes associated with PI3K-Akt/FoxO signaling axis (*upper network*) or cell adhesion (*lower network*)

Inorder to elucidate the biological processes which could be potentially regulated by miR-148a and miR-625-3p, miRNA target analysis was performed. The analysis identified 502 and 98 genes theoretically predicted as miR-148a and miR-625-3p targets (Fig. 4a). However, only 11 of them were determined as regulated in common between the semiRNAs (Fig. 4b). In addition, the analysis revealed that 29 target genes were associated with miR-148a with high experimental confidence. Further, to investigate any relations between miR-148a/miR-625-3p and epithelial-mesenchymal transition (EMT), the list of all known EMT genes retrieved from dbEMT was compared with the list of obtained target genes. Venn diagram analysis indicated that 13 and 6 EMT genes could be potentially regulated by miR-148a and miR-625-3p, respectively (Fig. 4c). Interestingly, among them key players of EMT ZEB1 and TGFB2 were predicted targets of miR-148a, and TGFBR1 – miR-625-3p. The list of EMT genes potentially regulated by these two miRNAs is depicted in Additional file 1: Table S1. Next, KEGG pathway enrichment analysis indicated 7 KEGG categories enrichedin miR-148a target-genes (Additional file 2: Table S2). Among them FoxO (*p* = 0.012) and PI3K-Akt (*p* = 0.0187) signaling pathways were the most significantly enriched functional categories. Inaddition, the pathway enrichment data revealed that categories associated with cell adhession including focal adhesion, regulation of actin cytoskeleton and ECM-receptor interaction were significantly enriched in miR-148a target-genes. miR-148a and target gene networks related to FoxO/PI3K-Akt (27 target-genes) and cell adhesion (21 target-genes) functional groups are visualized in Fig. 4d. However, KEGG analysis did not obtain any significant pathway enrichment of miR-625-3p target-genes (data is not shonwn).

## Discussion

Multiple data affirm that miRNAs are involved in mechanisms of excessive growth, resistance to apoptosis, angiogenesis, invasion, and metastasis [27, 28]. In this study, we have investigated miRNA-148a and miRNA-625-3p expression, due to the growing evidence of their importance in carcinogenesis and progression of CRC as well as in prediction of treatment efficacy.

MiRNA-148a down-regulation has been detected in gastric, breast, non-small cell lung cancer and other tumors [20, 29, 30] compared to normal tissues. Several trials reported down-regulation of miRNA-148a in CRC cells and it’s association with more advanced disease and poor prognosis [18, 31–33].

Y. Hibino et al. [31] showed significant downregulation of miRNA-148a expression in CRC and adenoma compared to normal colonic mucosa. MiRNA-148a expression was significantly lower in high-grade adenoma compared to low grade (*p* = 0.005). In more advanced CRC casesit differed depending on pT (*p* = 0.010), pN (*p* = 0.007) and stage (*p* = 0.027). Furthermore, a low miRNA-148a expression was an independent prognostic factor of OS for stage III patients (HR 4.421, 95% CI 1.473–18.428, *p* = 0.006).

H. L. Tsai et al. [32] found a relationship between early relapse of CRC patients in the II and III stages that underwent curative resection and low miRNA-148a expression in the tumor (OR 5.221; 95% CI, 2.068–13.174, *p* < 0.0001) and serum (*p* = 0.045). Lower miRNA-148a expression in the tumor was also associated with shorter DFS (*p* = 0.0006) and a worse OS (*p* = 0.0156). Investigators demonstrated that miRNA-148a inhibits cell proliferation (possibly through the mechanism of arresting the cell cycle during phase 2) and migration.

M. Takahashi et al. [18] reported that low miRNA-148a expression is associated with advanced CRC stages (III/IV versus normal mucosa, *p* < 0.0001) and is an independent prognostic factor of PFS for stage III patients (HR 1.83, 95% CI 1.12–2.99, *p* = 0.017) and OS for stage IV patients (HR 1.93, 95% CI 1.15–3.23, *p* = 0.014). In addition, they reported that miRNA-148a expression may be regulated by promoter methylation, and that there is an independent association between miRNA-148a methylation and poor survival of stage IV CRC patients.

H. Huang et al. [34] reported that MiRNA-148a negatively regulates the expression of MMP7, which is involved in tumor cell invasion and associated with advanced stages and poor clinical outcome of CRC.

H. Zhang et al. [35], however, did not show any significant differences between miRNA-148a expression in tumor and normal tissue, as well as no correlation with patients’ clinical and pathological characteristics. It was confirmed that BCL-2 is a target of miRNA-148a, revealing its proapoptotic potential.

Also there is evidence regarding the importance in cancer development of other targets of miRNA-148a, such as *DNMT1* – inhibition of which increases expression of multiple genes by epigenetic changes (reduced methylation) [36]; ERBB3 – involved in angiogenesis [37]; *CDC25B* – cell cycle regulator [38]; and *IGF-IR, AKT, MAPK/ERK* – cell growth [39]. By silencing these and potentially other target genes, miRNA-148a inhibits cell growth, proliferation, angiogenesis, invasion, andsusceptibility to apoptosis.

In the present study, we determined the deregulation of miRNA-148a in tumor tissue, compared to normal colonic mucosa. There was a trend toward higher miRNA-148a expression in mucinous adenocarcinoma compared to adenocarcinoma (*p* = 0.068), but there were no associations with other major clinical and pathological features (such as age, gender, tumor location and size, lymph node involvement, number or site of metastases, KRAS mutation status). These findings correspond to the results of the H. Zhang study [35], but in both studies, the results might have been influenced by the small sample size.

In our study, miRNA-148a expression was lower in cancers with tumor budding (median values accordingly 0.314 and 0.753, *p* = 0.011), which has been found by other studies to be associated with a more invasive phenotype and distant metastases [40] and determined as prognostic factor independent from CRC stage [3, 4].

Tumor budding indicates the presence of individual cells and small clusters of tumor cells at the invasive front of the carcinoma and is thought to represent epithelial-mesenchymal transition (EMT) [3, 4]. EMT is mainly regulated by SNAIL, TWIST and ZEB transcription factors and characterized by down-regulation of epithelial markers (E-cadherin, claudin etc.) and up-regulation of mesenchymal markers (Fibronectin, N-cadherin, MMPs etc.) [40]. The correlation between EMT and tumor budding is based on similarities in characteristics – low membranous level of E-cadherin [41], upregulation of fibronectin and vimetin [42], activation of TGF-β [43] and other pathways [7].

Studies demonstrated that miRNA-148a suppresses EMT and the expression of several genes overexpressed in EMT [20, 21]. J.P. Zhang et al. [21] reported that miR-148a may promote the expression of an epithelial marker (E-cadherin), reduce the levels of mesenchymal markers (N-cadherin, fibronectin or vimentin), and negatively regulate MET/SNAIL signaling and in turn inhibit the EMT and metastasis of hepatoma cells. Z. Qin et al. [22] recently suggested that miR-148a, miR-505 and miR-1207-5p might be induced by growth factors, and function as EMT and metastasis inhibitors by suppressing key EMT and WNT signaling molecules.

MiRNA-625-3p is another miRNAwith growing evidence of involvement in development and progression of different types of cancer [44–47]. M. Wang et al. [45] have reported that miRNA-625-3p silences the expression of integrin-linked kinase (ILK), the activation of which results in oncogenic transformation to invasive and metastatic phenotypes. In concordance with this, an inverse relationship has been established between downregulated miRNA-625-3p expression in gastric cancer and lymph node metastasis. In recent studies, downregulation of miRNA-625-3p was associated with more advanced Tstages and metastases of esophageal cancer. In addition, it was demonstrated that miRNA-625-3p negatively regulates Sox2, which promotes tumor invasion and metastasis by stimulating EMT via regulation of WNT/β-catenin, and tumor growth via AKT/MTOR signaling pathways [45, 47].

The role of miRNA-625-3p in CRC is not well understood. X. Lou et al. [46] reported that decreased miRNA-625-3p expression correlated with lymphnode metastasis (*p* = 0.038), liver metastasis (*p* = 0.031), and was an independent prognostic factor for OS (HR 0.454; CI 95% 0.218–0.943, *p* = 0.034). H. Zheng et al. [48] showed that miRNA-625-3p might positively regulate colorectal cancer cell migration and invasion through the SCAI/E-cadherin/MMP9 pathway. M. H. Rasmussen et al. [17] did not ascertain a prognostic significance between miRNA-625-3p expression and CRC stage II-III, but demonstrated that a high level of miRNA-625-3p is associated with shorter OS (HR 1.87, CI 95% 1.2–3.41, *p* = 0.039) in metastatic CRC.

In our study, we found a significantly lower miRNA-625-3p expression in tumors with budding phenomenon (0.404 and 0.620 respectfully, *p* = 0.036). This finding indicates potential miRNA-625-3p involvement in EMT, which is also supported by earlier reported data about this miRNAs role in WNT/β-catenin and SCAI/E-cadherin/MMP9 pathways.

Taking in to account the wide spectrum of miRNA targets, they have been actively investigated as potential predictive markers. One of the main chemotherapy agents for treating CRC – oxaliplatin –induces cell apoptosis by causing DNA interstrand cross-links, [49]. Bcl2 as target of miRNA-148a provides the rationale for using miRNA-148aas a predictive marker of the response to oxaliplatin. M. Takahashi et al. [18] reported a significant association between low miRNA-148a expression and a worse response to oxaliplatin-based therapy (*p* = 0.006), a worse OS (median 16.1 vs. 25.6 month, *p* = 0.024), and a trend toward a worse PFS (median 8.1 vs. 10.1 month, *p* = 0.16) in metastatic CRC patients. In contrast, J.B. Kjersem et al. [11] reported an association between high miRNA-148a expression in the plasma of metastatic CRC patients receiving oxaliplatin-based chemotherapy and a decrease in PFS (HR 1.3; 95% CI 1.1–1.6, *p* = 0.007). M. H. Rasmussen et al. [19] have reported that high miRNA-625-3p expression is a negative predictive factor for response to first line oxaliplatin-based chemotherapy in metastatic CRC (OR 6.25, 95% CI 1.8–21.0). Current evidence suggests that the investigation of both miRNAs mentioned, could be beneficial in predicting the efficacy of oxaliplatin. Our results did not confirm this statement – neither miRNA-148a expression nor miRNA-625-3p expression was associated with response rates in studied patients. High miRNA-148a expression was associated with worse PFS (6 month and 9 month respectively, *p* = 0.033), but not OS (18 month and 24 month respectively, *p* = 0.199). MiRNA-625-3p did not influence PFS or OS. Small sample size of the study limited the statistical power to identify differences in PFS and OS.

To our knowledge, our study is the first that determined an association between low miRNA-148a and miRNA-625-3p expression and tumor budding. The small sample size is the major limitation of our study and our findings need to be validated in an independent cohort. However, our results allow us to propose an interesting hypothesis regarding the possible role of the tested RNAs in the tumor budding process which is likely associated with EMT.

Our bioinformatics analysis revealed that 19 EMT related genes including key regulators of EMT – ZEB1, TGFB2 and TGFBR1 – are potential target genesof miRNA-148a and miRNA-625-3p, supporting the roles of these miRNAs as regulators of EMT. In addition, our results are consistent with published data, regarding the role of miRNA-148a and miRNA-625-3p in EMT regulation.

## Conclusions

The presented study has identified a significant relationship between low miRNA-148a and miRNA-625-3p expression and tumor budding, which is thought to represent EMT. The results show that the studied miRNAs may be associated with a more aggressive phenotype and could be the potential prognostic and predictive biomarkers in CRC. Further investigation is needed to confirm the involvement these miRNAs in EMT, and their prognostic and predictive value.

## Additional files

Additional file 1: Table S1. Full list of genes associated with EMT which could be potentially regulated by miR-148a or miR-625-3p miRNAs. (DOCX 11 kb)

Additional file 2: Table S2. Full list of KEGG pathway categories enriched in miR-148a target-genes. (DOCX 12 kb)

## Abbreviations

CRC: Colorectal cancer
EMT: Epithelial-mesenchymal transition
miRNA, miR: micro RNA
OS: Overall survival
PFS: Progression free survival

## References

[1] Jemal A, Bray F, Center MM, Ferlay J, Ward E, Forman D (2011). Global cancer statistics. CA Cancer J Clin.

[2] Van Cutsem E, Kohne CH, Hitre E, Zaluski J, Chien CRC, Makhson A (2009). Cetuximab and Chemotherapy as Initial Treatment for Metastatic Colorectal Cancer. New England Journal of Medicine.

[3] Labianca R, Nordlinger B, Beretta GD, Mosconi S, Mandala M, Cervantes A (2013). Early colon cancer: ESMO clinical practice guidelines for diagnosis, treatment and follow-up. Ann Oncol.

[4] Graham RP, Vierkant RA, Tillmans LS, Wang AH, Laird PW, Weisenberger DJ (2015). Tumor budding in colorectal carcinoma confirmation of prognostic significance and histologic cutoff in a population-based cohort. Am J Surg Pathol.

[5] Koelzer VH, Zlobec I, Lugli A (2016). Tumor budding in colorectal cancer-ready for diagnostic practice?. Hum Pathol.

[6] Dawson H, Lugli A (2015). Molecular and pathogenetic aspects of tumor budding in colorectal cancer. Front Med (Lausanne).

[7] Grigore AD, Jolly MK, Jia DY, Farach-Carson MC, Levine H (2016). Tumor Budding: The Name is EMT. Partial EMT. Journal of Clinical Medicine.

[8] Bartel DP (2004). MicroRNAs: Genomics, biogenesis, mechanism, and function. Cell.

[9] Okugawa Y, Toiyama Y, Goel A (2014). An update on microRNAs as colorectal cancer biomarkers: where are we and what's next?. Expert Rev Mol Diagn.

[10] Hong L, Han Y, Yang JJ, Zhang HW, Zhao QC, Wu KC (2014). MicroRNAs in gastrointestinal cancer: prognostic significance and potential role in chemoresistance. Expert Opin Biol Ther.

[11] Schetter AJ, Leung SY, Sohn JJ, Zanetti KA, Bowman ED, Yanaihara N (2008). MicroRNA expression profiles associated with prognosis and therapeutic outcome in colon adenocarcinoma. JAMA.

[12] Volinia S, Calin GA, Liu CG, Ambs S, Cimmino A, Petrocca F (2006). A microRNA expression signature of human solid tumors defines cancer gene targets. Proc Natl Acad Sci U S A.

[13] Luo XY, Burwinkel B, Tao S, Brenner H (2011). MicroRNA signatures: novel biomarker for colorectal cancer?. Cancer Epidemiol Biomark Prev.

[14] Bandres E, Cubedo E, Agirre X, Malumbres R, Zarate R, Ramirez N (2006). Identification by Real-time PCR of 13 mature microRNAs differentially expressed in colorectal cancer and non-tumoral tissues. Molecular Cancer.

[15] Stiegelbauer V, Perakis S, Deutsch A, Ling H, Gerger A, Pichler M (2014). MicroRNAs as novel predictive biomarkers and therapeutic targets in colorectal cancer. World J Gastroenterol.

[16] Hur K, Toiyama Y, Schetter AJ, Okugawa Y, Harris CC, Boland CR, et al. Identification of a Metastasis-Specific MicroRNA Signature in Human Colorectal Cancer. J Natl Cancer Inst. 2015;107(3) doi:10.1093/jnci/dju492. 10.1093/jnci/dju492 PMC4334826 25663689

[17] Kjersem JB, Ikdahl T, Lingjaerde OC, Guren T, Tveit KM, Kure EH (2014). Plasma microRNAs predicting clinical outcome in metastatic colorectal cancer patients receiving first-line oxaliplatin-based treatment. Mol Oncol.

[18] Takahashi M, Cuatrecasas M, Balaguer F, Hur K, Toiyama Y, Castells A et al. The Clinical Significance of MiR-148a as a Predictive Biomarker in Patients with Advanced Colorectal Cancer. Plos One. 2012;7(10). doi:ARTN e46684. doi:10.1371/journal.pone.0046684. 10.1371/journal.pone.0046684 PMC3463512 23056401

[19] Rasmussen MH, Jensen NF, Tarpgaard LS, Qvortrup C, Romer MU, Stenvang J (2013). High expression of microRNA-625-3p is associated with poor response to first-line oxaliplatin based treatment of metastatic colorectal cancer. Mol Oncol.

[20] Xue J, Chen Z, Gu X, Zhang Y, Zhang W (2016). MicroRNA-148a inhibits migration of breast cancer cells by targeting MMP-13. Tumour Biol.

[21] Zhang JP, Zeng C, Xu L, Gong J, Fang JH, Zhuang SM (2014). MicroRNA-148a suppresses the epithelial-mesenchymal transition and metastasis of hepatoma cells by targeting met/Snail signaling. Oncogene.

[22] Qin Z, He W, Tang J, Ye Q, Dang W, Lu Y (2016). MicroRNAs provide feedback regulation of epithelial-Mesenchymal transition induced by growth factors. J Cell Physiol.

[23] Horcic M, Koelzer VH, Karamitopoulou E, Terracciano L, Puppa G, Zlobec I (2013). Tumor budding score based on 10 high-power fields is a promising basis for a standardized prognostic scoring system in stage II colorectal cancer. Hum Pathol.

[24] Paraskevopoulou MD, Georgakilas G, Kostoulas N, Vlachos IS, Vergoulis T, Reczko M et al. DIANA-microT web server v5.0: service integration into miRNA functional analysis workflows. Nucleic Acids Research. 2013;41(Web Server issue):W169-WW73. doi:10.1093/nar/gkt393. 10.1093/nar/gkt393 PMC3692048 23680784

[25] Chou C-H, Chang N-W, Shrestha S, Hsu S-D, Lin Y-L, Lee W-H (2016). miRTarBase 2016: updates to the experimentally validated miRNA-target interactions database. Nucleic Acids Res.

[26] Wang J, Duncan D, Shi Z, Zhang B (2013). WEB-based GEne SeT AnaLysis toolkit (WebGestalt): update 2013. Nucleic Acids Res.

[27] Hanahan D, Weinberg RA (2011). Hallmarks of cancer: the next generation. Cell.

[28] Hausser J, Zavolan M. Identification and consequences of miRNA-target interactions-beyond repression of gene expression. Nature Reviews Genetics. 2014;vol 15:pg 599. 2014;15(10):702. 10.1038/nrg3765 25022902

[29] Sakamoto N, Naito Y, Oue N, Sentani K, Uraoka N, Oo HZ (2014). MicroRNA-148a is downregulated in gastric cancer, targets MMP7, and indicates tumor invasiveness and poor prognosis. Cancer Sci.

[30] Joshi P, Jeon YJ, Lagana A, Middleton J, Secchiero P, Garofalo M (2015). MicroRNA-148a reduces tumorigenesis and increases TRAIL-induced apoptosis in NSCLC. Proc Natl Acad Sci U S A.

[31] Hibino Y, Sakamoto N, Naito Y, Goto K, Oo HZ, Sentani K (2015). Significance of miR-148a in colorectal Neoplasia: Downregulation of miR-148a contributes to the carcinogenesis and cell invasion of colorectal cancer. Pathobiology.

[32] Tsai HL, Yang IP, Huang CW, Ma CJ, Kuo CH, Lu CY (2013). Clinical significance of microRNA-148a in patients with early relapse of stage II stage and III colorectal cancer after curative resection. Transl Res.

[33] Chen Y, Song Y, Wang Z, Yue Z, Xu H, Xing C (2010). Altered expression of MiR-148a and MiR-152 in gastrointestinal cancers and its clinical significance. J Gastrointest Surg.

[34] Huang Y, Yu HJ, Lei H, Xie CH, Zhong YH. Matrix metalloproteinase 7 is a useful marker for 5-fluorouracil-based adjuvant chemotherapy in stage II and stage III colorectal cancer patients. Medical Oncology. 2014;31(3) doi:10.1007/S12032-013-0824-0. 10.1007/s12032-013-0824-0 24469951

[35] Zhang H, Li Y, Huang Q, Ren X, Hu H, Sheng H (2011). MiR-148a promotes apoptosis by targeting Bcl-2 in colorectal cancer. Cell Death Differ.

[36] Zhan Q, Fang Y, Deng XX, Chen H, Jin JB, Lu XX (2015). The interplay between miR-148a and DNMT1 might be exploited for pancreatic cancer therapy. Cancer Investig.

[37] Yu J, Li Q, Xu Q, Liu L, Jiang B (2011). MiR-148a inhibits angiogenesis by targeting ERBB3. J Biomed Res.

[38] Liffers ST, Munding JB, Vogt M, Kuhlmann JD, Verdoodt B, Nambiar S (2011). MicroRNA-148a is down-regulated in human pancreatic ductal adenocarcinomas and regulates cell survival by targeting CDC25B. Lab Investig.

[39] Chen Y, Song YX, Wang ZN. The MicroRNA-148/152 Family: Multi-faceted Players. Molecular Cancer. 2013;12 doi:10.1186/1476-4598-12-43. 10.1186/1476-4598-12-43 PMC3671164 23683438

[40] De Smedt L, Palmans S, Sagaert X (2016). Tumour budding in colorectal cancer: what do we know and what can we do?. Virchows Arch.

[41] Bronsert P, Enderle-Ammour K, Bader M, Timme S, Kuehs M, Csanadi A (2014). Cancer cell invasion and EMT marker expression: a three-dimensional study of the human cancer-host interface. J Pathol.

[42] Masugi Y, Yamazaki K, Hibi T, Aiura K, Kitagawa Y, Sakamoto M (2010). Solitary cell infiltration is a novel indicator of poor prognosis and epithelial-mesenchymal transition in pancreatic cancer. Hum Pathol.

[43] Jensen DH, Dabelsteen E, Specht L, Fiehn AMK, Therkildsen MH, Jonson L (2015). Molecular profiling of tumour budding implicates TGF-mediated epithelial-mesenchymal transition as a therapeutic target in oral squamous cell carcinoma. J Pathol.

[44] Wang M, Li CL, Nie H, Lv X, Qu Y, Yu BQ (2012). Down-regulated miR-625 suppresses invasion and metastasis of gastric cancer by targeting ILK. FEBS Lett.

[45] Wang ZQ, Qiao Q, Chen M, Li XH, Wang ZJ, Liu CX (2014). miR-625 down-regulation promotes proliferation and invasion in esophageal cancer by targeting Sox2. FEBS Lett.

[46] Lou XL, Qi XL, Zhang Y, Long HD, Yang JJ (2013). Decreased expression of microRNA-625 is associated with tumor metastasis and poor prognosis in patients with colorectal cancer. J Surg Oncol.

[47] Li C, Li DC, Che SS, Ma K, Wang YJ, Xia LH (2015). The decreased expression of miR-625 predicts poor prognosis of esophageal squamous cell carcinoma. Int J Clin Exp Med.

[48] Zheng HL, Ma RQ, Wang QZ, Zhang P, Li DP, Wang QW (2015). MiR-625-3p promotes cell migration and invasion via inhibition of SCAI in colorectal carcinoma cells. Oncotarget.

[49] Lavarino C, Pilotti S, Oggionni M, Gatti L, Perego P, Bresciani G (2000). p53 gene status and response to platinum/paclitaxel-based chemotherapy in advanced ovarian carcinoma. J Clin Oncol.

