# Supplementary material for: Down-regulation of miRNA-148a and miRNA-625-3p in colorectal cancer is associated with tumor budding
Source: BMC Cancer. 2017 Sep 1;17:607. doi: 10.1186/s12885-017-3575-z (PMC5580437; doi:10.1186/s12885-017-3575-z)
Supplement: Supplementary file 1 — Full list of genes associated with EMT which could be potentially regulated by miR-148a or miR-625-3p miRNAs. (DOCX 11 kb) [file 12885_2017_3575_MOESM1_ESM.docx]

**Supplementary table 1.** Full list of genes associated with EMT which could be potentially regulated by miR-148a or miR-625-3p miRNAs.

| **miRNA** | **Number of genes** | **Genes** |
| --- | --- | --- |
| **miR-148a** | 13 | EPAS1; ZEB1; MAP3K4; CDKN1B; CUX1; TGFA; PAG1; ROCK1; FLT1; EPB41L5; PTPN14; TGFB2; ADAM17; |
| **miR-625-3p** | 6 | ZBTB33;TGFBR1; KRAS;HMGA2; HMGB3; MKL2. |
