# Supplementary material for: Down-regulation of miRNA-148a and miRNA-625-3p in colorectal cancer is associated with tumor budding
Source: BMC Cancer. 2017 Sep 1;17:607. doi: 10.1186/s12885-017-3575-z (PMC5580437; doi:10.1186/s12885-017-3575-z)
Supplement: Supplementary file 2 — Full list of KEGG pathway categories enriched in miR-148a target-genes. (DOCX 12 kb) [file 12885_2017_3575_MOESM2_ESM.docx]

**Supplementary table 2.** Full list of KEGG pathway categories enriched in miR-148a target-genes.

| **Category** | **Number of genes** | **Genes** | **p value** |
| --- | --- | --- | --- |
| **FoxO signaling pathway** | 13 | BCL2L11; CDKN1B; GADD45A; S1PR1; IGF1; NRAS; PRKAA1; SOS1; SOS2; TGFB2; USP7; PIK3R3; HOMER1. | 0.012 |
| **PI3K-Akt signaling pathway** | 21 | BCL2L11; CDKN1B; COL2A1; COL4A1; CSF1; ITGA11; FLT1; IGF1; ITGA5; ITGA9; ITGB8; LAMA4; LAMB2; NRAS; PPP2R5E; PRKAA1; SOS1; SOS2; TEK; YWHAB; PIK3R3. | 0.0187 |
| **Focal adhesion** | 15 | COL2A1; COL4A1; ITGA11; FLT1; IGF1; ITGA5; ITGA9; ITGB8; LAMA4; LAMB2; PPP1CB; ROCK1; SOS1; SOS2; PIK3R3. | 0.0187 |
| **Hypertrophic cardiomyopathy (HCM)** | 9 | ITGA11; IGF1; ITGA5; ITGA9; ITGB8; ATP2A2; PRKAA1; SGCB; TGFB2. | 0.0187 |
| **Renal cell carcinoma** | 8 | EPAS1; NRAS; SLC2A1; SOS1; SOS2; TGFA; TGFB2; PIK3R3 | 0.0187 |
| **Regulation of actin cytoskeleton** | 15 | NCKAP1; ITGA11; MRAS; ARHGEF12; APC; ITGA5; ITGA9; ITGB8; NRAS; PPP1CB; ROCK1; SOS1; SOS2; PIK3R3; WASL. | 0.0232 |
| **ECM-receptor interaction** | 8 | COL2A1; COL4A1; ITGA11; ITGA5; ITGA9; ITGB8; LAMA4; LAMB2. | 0.05 |
